# Exchange Reactions Between Albumin-Gold(I)-Triethylphosphine and Me_3_PAuCl or iPr_3_PAuCl_3_ Studied by ^13^P NMR Spectroscopy

**DOI:** 10.1155/MBD.1994.516

**Published:** 1994

**Authors:** Anvarhusein A. Isab, C. Frank Shaw III, Amalia Munoz

**Affiliations:** 1 Department of Chemistry University of Wisconsin-Milwaukee Milwaukee WI 53201-0413 USA


					EXCHANGE REACTIONS
BETWEEN ALBUMIN-GOLD(1)-TRIETHYLPHOSPHINE AND
Me3PAuCI OR iPr3PAuCI STUDIED BY 3p NMR SPECTROSCOPY
Anvarhusein A. Isab, C. Frank Shaw III and Amalia Munoz
Department of Chemistry, University of Wisconsin-Milwaukee, Milwaukee, Wl 53201-0413, USA
The AIbSAuPEt3 complex was prepared in vitro by reacting albumin (AIbSH) with aumnofin
(Et3PAuSATg). The Et3PAu+ entity was found to be labile to exchange in the presence f
Me3PAuCI and iPraPAuCI. 31p NMR spectroscopy was used to follow these exchange reactions.
Either Me3PAu+ or iPr3PAu+ replaces Et3PAu+ from AIbSAuPEt3 complex. Et3PAu+ and
R3PAu+ (R Me, iPr) were both bound to the weak binding sites (principally histidine) f the
protein. Since the iPr3P ligand is bulkier than MeaPAu+ (reflected by its bigger Tollman cone
angle) it is surprising that it replaces Et3PAu+ almost equally as well as Me3PAu+.
We also demonstrate that Et3PAu+ bound to the weak (primarily histidine) binding sites of
Cys-34-modified albumin ((Et3PAU)xAIbS-Acm, P ca. 28 ppm) can be transferred to the Cys-34
of AIbSAuPEt3 to form the bis complex AIbS(AuPEt3)2 (P 35.7 ppm). The formation of
AIbS(AuPEt3)2 shows that the bis complex is indeed in equilibrium with the weak-binding sites
characterized by P 28 ppm. The transfer of Et3PAu+ between protein species is relevant to
mechanisms by which gold may be transferred between proteins in vivo.
516

				

